# A Standardized Method for the Construction of Tracer Specific PET and SPECT Rat Brain Templates: Validation and Implementation of a Toolbox

**DOI:** 10.1371/journal.pone.0122363

**Published:** 2015-03-30

**Authors:** David Vállez Garcia, Cindy Casteels, Adam J. Schwarz, Rudi A. J. O. Dierckx, Michel Koole, Janine Doorduin

**Affiliations:** 1 Department of Nuclear Medicine and Molecular Imaging, University of Groningen, University Medical Center Groningen, Groningen, The Netherlands; 2 Molecular Small Animal Imaging Center (MoSAIC), KU Leuven, Leuven, Belgium; 3 Department of Psychological and Brain Sciences, Indiana University, Bloomington, Indiana, United States of America; INSERM U894, France

## Abstract

High-resolution anatomical image data in preclinical brain PET and SPECT studies is often not available, and inter-modality spatial normalization to an MRI brain template is frequently performed. However, this procedure can be challenging for tracers where substantial anatomical structures present limited tracer uptake. Therefore, we constructed and validated strain- and tracer-specific rat brain templates in Paxinos space to allow intra-modal registration. PET [^18^F]FDG, [^11^C]flumazenil, [^11^C]MeDAS, [^11^C]PK11195 and [^11^C]raclopride, and SPECT [^99m^Tc]HMPAO brain scans were acquired from healthy male rats. Tracer-specific templates were constructed by averaging the scans, and by spatial normalization to a widely used MRI-based template. The added value of tracer-specific templates was evaluated by quantification of the residual error between original and realigned voxels after random misalignments of the data set. Additionally, the impact of strain differences, disease uptake patterns (focal and diffuse lesion), and the effect of image and template size on the registration errors were explored. Mean registration errors were 0.70±0.32mm for [^18^F]FDG (*n* = 25), 0.23±0.10mm for [^11^C]flumazenil (*n* = 13), 0.88±0.20 mm for [^11^C]MeDAS (*n* = 15), 0.64±0.28mm for [^11^C]PK11195 (*n* = 19), 0.34±0.15mm for [^11^C]raclopride (*n* = 6), and 0.40±0.13mm for [^99m^Tc]HMPAO (*n* = 15). These values were smallest with tracer-specific templates, when compared to the use of [^18^F]FDG as reference template (*p*&0.001). Additionally, registration errors were smallest with strain-specific templates (*p*&0.05), and when images and templates had the same size (*p*≤0.001). Moreover, highest registration errors were found for the focal lesion group (*p*&0.005) and the diffuse lesion group (*p = n*.*s*.). In the voxel-based analysis, the reported coordinates of the focal lesion model are consistent with the stereotaxic injection procedure. The use of PET/SPECT strain- and tracer-specific templates allows accurate registration of functional rat brain data, independent of disease specific uptake patterns and with registration error below spatial resolution of the cameras. The templates and the SAMIT package will be freely available for the research community.

## Introduction

Nuclear medicine imaging techniques are increasingly used for the study of rodent models of a variety of human brain diseases. The use of these functional images allows the researcher to measure physiological processes, biochemical pathways and neurotransmitters in vivo. The ability to perform longitudinal, within-animal scans greatly facilitates the investigation of chronic diseases and the evaluation of neuropharmacological interventions. However, the resolution that can be obtained in current small animal positron emission tomography (PET) and single-photon emission computed tomography (SPECT) scanners is a limiting factor during the analysis. Therefore, the optimal use of the imaging data becomes crucial.

A powerful and widely-used approach for the analysis of neuroimaging data is based on the adoption of a common reference space to which images from individual subjects and time points are spatially normalized [1]. This allows direct within- or between-subject comparisons and the application of standard, pre-defined reference maps and masks, including atlas structures. However, the normalization of functional images without its accompanying, simultaneous acquired, structural image is challenging due to the tracer specific spatial profiles and tracer dependent amount of anatomical reference points. The availability of tracer specific templates aligned in a standard reference space would enable the use of automatic normalization of functional images to a template, therefore minimizing the user dependent variability and providing direct access to corresponding anatomical atlases and reference coordinates. Moreover group comparisons could be performed using a voxel-based and/or VOI-based (volume of interest) analysis.

The aim of the current study was to standardize the methodology for the construction of rat brain PET and SPECT tracer specific templates, and to provide and share tools necessary for this procedure and for the subsequent voxel-based and/or VOI-based analysis. The steps for the construction of the templates were based on previous work of Casteels et al. [2,3] but revised to obtain symmetrical templates and extended to other PET and SPECT specific tracers, including [^18^F]FDG for the assessment of functional metabolism, [^11^C]flumazenil for GABA_A_ receptors, [^11^C]MeDAS for myelin integrity, [^11^C]PK11195 for microglia activation, [^11^C]raclopride for D_2/3_ dopamine receptors, and [^99m^Tc]HMPAO for the measurement of cerebral blood flow. In addition, a more recent T_2_-weighted MRI template in Paxinos space [4] was used as reference dataset. For the spatial normalization of brain data of healthy animals, the added value of tracer and strain specific templates was evaluated and compared to the more standard and commonly available [^18^F]FDG template of the rat brain, an aspect that has not been addressed so far in a preclinical setting. Moreover, the relevance of strain specific templates was determined by comparing the registration errors of [^18^F]FDG and [^11^C]PK11195 brain PET scans of both healthy Sprague-Dawley and Wistar rats using either a strain specific or a general template. And finally, the effect in the registration errors of focal and diffuse alterations of uptake was explored with [^11^C]PK11195 images.

In addition, we present a software package that works as an extension of SPM (Wellcome Department of Cognitive Neurology, University College London, UK): SAMIT (Small Animal Molecular Imaging Toolbox). The aim of this toolbox is to facilitate the construction of new tracer specific templates and the subsequent voxel-based analysis of small animal PET and SPECT brain images. In human studies, the analysis of functional neuroimaging data is frequently performed with the SPM software developed by Friston et al. Although some studies have used this software in a preclinical setting, there was not an easy to use approach widely available. Some efforts have been made to allow the use of SPM in the study of the rat brain images. One of the first extensions came with the distribution of a MRI rat brain template from the Karolinska Institute [5], developed for the SPM99 version, released in January 2000. This toolbox is not functional anymore with the newest versions of SPM (SPM8 and SPM12), but its MRI rat template was widely spread into the scientific community. More recently, Nie et al. [6] published another rat brain MRI template accompanied with a SPM toolbox, compatible with SPM8 (released in April 2009). In that toolbox, the MRI template used for spatial normalization of the data was not oriented into the standard Paxinos space [7] and the anterior commissure was adopted as the center of coordinates, while the bregma is the standard reference in the rat brain coordinates system. Moreover, several transformations are performed to the image during the process of analysis, what makes the exchange of the scans with other software packages or the interpretation of the results outside the framework of the toolbox difficult. Therefore, we decided to develop a toolbox producing minimal changes to the original SPM code, compatible with the most recent versions of SPM. To the best of our knowledge, this is the first time that a toolbox of these characteristics is developed, focused on the analysis of small animal PET and SPECT functional brain images.

## Materials and Methods

### Animals

Functional brain data of male Sprague-Dawley rats (*n* = 30, weight of 329±48 [261–424] grams) and male Wistar rats (*n* = 107, weight of 291±47 [222–437] grams) were acquired from Harlan (Lelystad, The Netherlands). After arrival, the animals were allowed to acclimatize for at least seven days. The rats were housed in Makrolon cages on a layer of wood shavings in a room with constant temperature (21±2°C) and 12 hour light-dark regime (light phase from 7:00–19:00 hours). Standard laboratory chow and water were available *ad libitum*. The distribution of the rats across the groups is summarized in Table 1. The Institutional Animal Care and Use Committee of the University of Groningen (The Netherlands) approved all experiments, and all applicable institutional and/or national guidelines for the care and use of animals were followed.

**Table 1:** Distribution of the rats across experimental groups.

				**Weight**			**Range**		
	**Strain**	**Group**	**N**	**Mean**	**±**	**SD**	**Min**	**-**	**Max**
**PET**									
[^18^F]FDG	Sprague-Dawley	Healthy	9	376	±	31	323	-	424
	Wistar	Healthy	25	318	±	57	247	-	437
[^11^C]Flumazenil	Wistar	Healthy	13	250	±	20	222	-	288
[^11^C]MeDAS	Wistar	Healthy	15	260	±	15	233	-	284
[^11^C]PK11195	Sprague-Dawley	Healthy	11	317	±	51	261	-	395
		Injection of saline	10	298	±	18	272	-	328
	Wistar	Healthy	19	319	±	49	225	-	399
		Herpes encephalitis	14	301	±	35	250	-	350
[^11^C]Raclopride	Wistar	Healthy	6	291	±	37	235	-	350
**SPECT**									
[^99m^Tc]HMPAO	Wistar	Healthy	15	266	±	13	244	-	290
***Total***	*Sprague-Dawley*		*30*	*329*	*±*	*48*	*261*	*-*	*424*
	*Wistar*		*107*	*291*	*±*	*47*	*222*	*-*	*437*

### Study design

The study was divided into three sections. In the first section, brain data of healthy rats were used for the construction of strain- and tracer- specific PET templates of [^18^F]FDG, [^11^C]flumazenil, [^11^C]MeDAS, [^11^C]PK11195, and [^11^C]raclopride, and for the construction of a SPECT [^99m^Tc]HMPAO template. The image data used for the construction of the templates was characterized in terms of intersubject variability and right-to-left asymmetry of the tracer distribution in the rat brain.

In the second section of the study, the feasibility of the templates was explored by quantitative evaluation of the registration errors, performing random misalignments of the brain data. Different aspects of the template characteristics were tested:

1. The effect of tracer specific templates was evaluated by comparing the registration errors obtained using a tracer specific template versus the results obtained using the commonly available [^18^F]FDG template of the rat brain.
2. The added value of strain specific templates was explored with [^18^F]FDG and [^11^C]PK11195 scans of Sprague-Dawley and Wistar rats. A comparison of the registration errors was performed using strain specific templates, template of the opposite strain, or a template that combines both strains.
3. The effect of the image and template size in the registration errors was explored using [^18^F]FDG and [^11^C]PK11195 images from Wistar rats. The ‘small images’ (96x120x96 slides) were adjusted to the skull size, by using the same size as the MRI template. The ‘large images’ had a broader field of view, which included extra cranial structures (150x150x150 slides). The templates were also constructed in these two sizes, and the registration errors were obtained from pairwise comparison.
4. The impact of disease uptake patterns on the normalization accuracy was also explored, using two different disease models:
  - *Focal lesion model*: The rats used for the focal lesion model were obtained from a previous study [8]. For the purpose of this manuscript, only the animals with saline injection were selected. A stereotaxic injection of saline was performed in the right corpus callosum and striatum, corresponding to the bregma coordinates −0.3 mm anteroposterior, 3 mm lateral, and −3, −4.2, −5 mm dorsoventral. [^11^C]PK11195 PET scans (Sprague-Dawley, *n* = 10) were performed at day 3 and day 7 days after injection.
  - *Herpes encephalitis model (HSE)*: This model was described in detail previously [9]. In short, rats were inoculated with the herpes simplex virus type 1 under slight isoflurane anesthesia (5% in medical air) by applying 100 μl of phosphate-buffered saline with 1x10^7^ plaque-forming units of virus into the nostrils. The rats (Wistar, *n* = 14) underwent a dynamic scan of 60 min with [^11^C]PK11195 at day 6 or day 7 after inoculation.

In the third and last section of the study, voxel-based analysis of the two previous disease models was performed to evaluate the use of the templates in combination with the SAMIT package. The focal lesion model was chosen to evaluate the accuracy in reporting the coordinates of a known inflammatory process induced by stereotaxic injection of saline in the rat brain, while the effect of a broader inflammatory process was explored with the HSE model.

### Tracer Synthesis

The synthesis of the PET tracers [^11^C]PK11195 ([*N*-methyl-^11^C](*R*)-1-(2-chlorophenyl)-*N*-(1-methylpropyl)-3-isoquinoline carboxamide) and [^11^C]MeDAS ([*N*-methyl-^11^C]-4,4′-diaminostilbene) was performed as described previously [9,10], with a specific activity >30 Gbq/μmol and >50 GBq/μmol respectively. [^18^F]FDG (2-deoxy-2-[^18^F]fluoro-d-glucose) was produced by the Hamacher method (nucleophilic fluorination reaction followed by deprotection), with a specific activity >10 GBq/μmol.

[^11^C]Flumazenil (ethyl 8-fluoro-5-methyl-6-oxo-5,6-dihydro-4H-benzo[f]imidazo[1,5-a][1,4]diazepine-3-carboxylate) was performed as described previously [11]. Briefly, [^11^C]methyltriflate was trapped at room temperature in the reaction vial containing 0.5 mg of desmethyl-flumazenil (ABX 1700.0001) dissolved in 300 uL of dry aceton with 10uL of NaOH 1M. After the trapping of [^11^C]methyltriflate was completed, the reaction mixture was heated at 60°C for 1 min. Then, 0.7 ml of HPLC eluent was added (23% of acetonitrile, and 77% of 25 mM NaH_2_PO_4_ at pH 3.5 in sterile water). The mixture was purified by HPLC (Waters μBondpak C18 125 column 10μm, 7.8 mm x 300 mm). The purified product was diluted in 85 ml water and passed over an Oasis HLB 1cc (30 mg Waters) cartridge. The cartridge was washed twice with 8 ml saline, and eluted with 0.75 ml of ethanol and 4.5 ml of saline. The product was sterilized over 0.22 μm LG filter and collected in a sterile vial. Specific activity was >20 GBq/μmol.

[^11^C]Raclopride (3,5-dichloro-N-((1-ethyl-2-pyrrolidinyl)-methyl)-2-hydroxy-6-methoxy-benzamide) was labeled by trapping [^11^C]methyl iodide [12] in a solution of 1 mg desmethylraclopride and 1.4 mg sodium hydroxide in 300 μl dimethylsulfoxide. The reaction mixture was allowed to react for 4 minute at 80°C. After the reaction, the product was purified by HPLC using a μBondapak C18 column (7.8 mm x 300 mm) with acetonitrile/10 mM H_3_PO_4_ (30/70) as the eluent (flow 5 ml/min). To remove the organic solvents from the product, the collected HPLC fraction (retention time 8 min) was diluted with 100 ml of water and passed through an Oasis HLB 200 mg cartridge. The cartridge was washed twice with 8 ml of water and subsequently eluted with 0.8 ml of 1% H_3_PO_4_ in ethanol and 8 ml of phosphate buffer (pH 7.2). The product was sterilized by filtration over a 0.20 μm Millex LG filter. Quality control was performed by HPLC, using a μBondapak C18 column (300 mm x 3.9 mm) with acetonitrile/10 mM H_3_PO_4_ (30/70) as the eluent at a flow of 1 ml/min, and radiochemical purity was always >95% and the specific activity >50 GBq/μmol.

The SPECT tracer [^99m^Tc]HMPAO ([[(3*RS*,3'*RS*)-3,3'-[(2,2-dimethyltrimethylene)diimino][di-2-butanone]dioximato](3-)-N,N',N'',N''']oxotechnetium, ^99m^Tc) was synthesized using Ceretec Kit (GE Healthcare B.V., The Netherlands), and cobalt chloride as a stabilizer, following a procedure previously described [13].

### Data Acquisition

All PET imaging acquisitions were performed with a microPET Focus 220 camera (Siemens Medical Solutions USA, Inc.), with rats in transaxial position and the heads in the field of view. All the rats were anesthetized with isoflurane at 5% in medical air for induction, and 1.5–2% for maintenance. For all the acquisitions, a transmission scan of 515 seconds was performed with a ^57^Co point source, for attenuation and scatter correction.

The data used in the present study was collected from previous experiments performed in the department. Differences in the acquisition protocol are described below:

*[* ^*18*^ *F]FDG scans*: the rats were slightly anesthetized and the tracer was injected intraperitoneally. Then, rats were returned to their home cage and allowed to recover from anesthesia. At 40 min after injection the rats were anesthetized and positioned in the camera, where an acquisition of a 30 min static scan was performed 45 min after tracer injection.

*[* ^*11*^ *C]Flumazenil scans*: the rats were anesthetized and the tail vein was cannulated for tracer injection. Rats were placed in the camera, and a 60 min dynamic PET scan was started simultaneously with the injection of the tracer over 1 min, using an automatic pump at speed of 1 ml/min.

*[* ^*11*^ *C]MeDAS*: the rats were anesthetized and directly positioned in the camera. Simultaneously with the injection of the tracer via de penile vein a dynamic scan of 60 min was started.

*[* ^*11*^ *C]PK11195*: the rats were scanned using three different protocols. In the first protocol, the rats were slightly anesthetized and intravenously injected via the penile vein. Then, the rats were returned to their home cage and allowed to recover from anesthesia. At 40 min after injection, the rats were anesthetized and positioned in the camera for a 30 min static acquisition, performed at 45 min after tracer injection. In the second protocol, the rats were anesthetized and positioned in the camera, where the tracer was intravenously injected via the penile vein, and a 60 min dynamic scan was started simultaneous with tracer injection. In the third protocol, the rats were first cannulated into the femoral vein after induction of anesthesia, and then positioned in the camera. The tracer was injected over 1 min, using an automatic pump at speed of 1 ml/min, and a 60 min dynamic scan was started.

*[* ^*11*^ *C]Raclopride*: rats were anaesthetized and directly positioned in the camera. Tracer was injected via the penile vein, and a 60 min dynamic PET scan was started simultaneously.

The [^99^Tc]HMPAO acquisitions were performed with a high-resolution focusing multi-pinhole SPECT system (U-SPECT-II, MILabs, The Netherlands). Rats were anesthetized with isoflurane and intravenously injected with [^99^Tc]HMPAO via the penile vein. Hereafter the rats were positioned in the small animal SPECT camera in transaxial position with the head in the field of view. An acquisition scan of 45 min was started at 15 min after tracer injection.

### Image Reconstruction

For both the 60 min dynamic PET scans and 30 min static PET scans, the list-mode data were reconstructed into a single frame representing the last 30 min of the scan. The emission data were iteratively reconstructed (OSEM2D, 4 iterations, 16 subsets) after being normalized and corrected for attenuation, scatter and decay. Final images had a 128x128x95 matrix with a pixel width of 0.475 mm and a slice thickness of 0.796 mm.[^99^Tc]HMPAO images were reconstructed using U-SPECT-Rec v1.34i3 (MILabs, The Netherlands) with a pixel-based ordered-subsets expectation maximum (POSEM) algorithm with 16 subsets and 6 iterations, resulting in a single frame of 45 min corrected for attenuation and scatter. Final images had a 123x123x195 matrix with a pixel width and slice thickness of 0.375 mm.

Voxel-wise parametric standardized uptake value (SUV) images were constructed for all the scans. For [^18^F]FDG and [^99^Tc]HMPAO the values were corrected for the mean uptake of the whole brain.

### Data Preparation

Each image scan was first manually aligned with the stereotaxic T_2_-weighted MRI template using VINCI 4.36 software (Max Planck Institute for Metabolism Research, Cologne, Germany). New images were cropped and resliced into a 180x180x180 matrix dimension. According to the Nyquist frequency, the dimensions of the voxel size was decided to be 0.2 mm; about half the size of the smallest pixel, i.e. 0.375 mm from the SPECT reconstructed images.

### Template Construction

**Fig 1:**
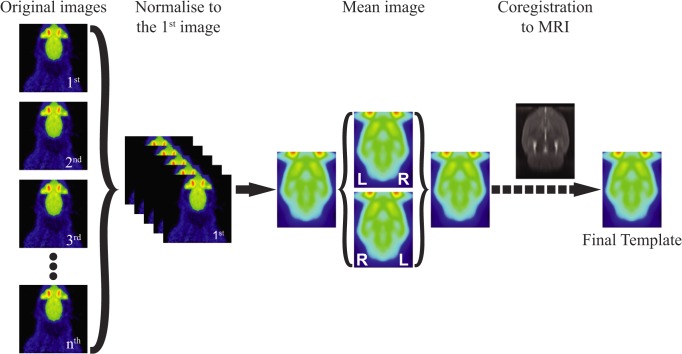
Flowchart. Construction of new tracer specific templates.

The procedure used for the construction of the templates was based on work by Casteels et al. [2,3] but revised to obtain symmetrical templates (Fig. 1). This process was automatized and implemented in SAMIT, using the functions included in SPM8 (Wellcome Department of Cognitive Neurology, University College London, UK), without the use of masked images during the procedure. The construction of the T_2_-weighted MRI template and its co-registration with the Paxinos anatomical atlas has been previously described [4]. The procedure for the construction of functional templates can be divided in three steps. First, one representative image of each set of tracer was selected as “standard” for that specific tracer. Then, each of the individual scans was normalised into the space of the representative one. This within modality affine registration was done by minimizing the sum of squares differences between the image which is to be normalized, and the reference image. Secondly, a symmetrical voxel-wise averaged template was obtained from the previously aligned images. For that, a flipped left-right duplicate of the previously obtained average image was created, and normalized into the original average template. And third, a cross-modality registration was performed between the symmetric averaged image and the reference MRI. This procedure was done using a rigid-body transformation based on the normalized mutual information maximization algorithm. Then, the transformation matrix obtained in the co-registration was applied to all the images used in the construction of the template, for further use in the study.

All rat brain image data which were spatially normalized to these functional templates were therefore positioned in the Paxinos stereotaxic coordinate system, facilitating the reporting of results and enabling the use of predefined standard-space atlas structures and other masks associated with the MRI template.

### Volumes of interest

A 3D volumetric atlas was constructed from the electronic version of the 78 coronal figures published in the 4^th^ edition of the Paxinos and Watson atlas, following the proposed framework of Majka et al. [14]. Composite structures were defined, as many of the individual structures are small relative to typical spatial resolution of PET and SPECT.

For the purpose of this study, the volumes of interest (VOI) were defined to represent the major cortical and subcortical structures of the rat brain, including nucleus accumbens, amygdala, caudate-putamen, cerebellum, cortex, globus pallidus, hippocampus, hypothalamus, medulla, midbrain, pons, septum and thalamus. Independent VOIs were obtained for left and right sides of the brain.

### Registration Error

The feasibility to register each individual scan to the selected template was quantified following a previously described procedure [2,3,15,16], which gives a realistic idea of the registration error, and was implemented in SAMIT for the evaluation of future templates. For the purpose of this study, all the images used in the construction of the templates, or used for the evaluation of the focal lesion and the HSE, were included in the analysis. Each of these images underwent 40 random misalignments: 10 translations, 10 rotations, 10 linear stretchings, and 10 combinations of the 3 previous parameters. The misalignments were generated with the uniformly distributed pseudorandom integer function, within −0.5 mm to +0.5 mm of translation, −20° to +20° of rotation, and −10% to +10% of linear stretching along the 3 orthogonal axes/planes. For the combined misalignment, rotation was defined within −10° to +10°. These values were based on typical magnitudes that can be found in realistic situations. Each resultant image volume was smoothed with a Gaussian kernel of 8mm and then registered again to the selected template with affine registration using least squares function. For each voxel (*x*,*y*,*z*) in the original image, the position after misalignment and posterior registration was computed. Then, the distance $\left(\sqrt{{\text{Δ}}_{x}^{2}+{\text{Δ}}_{y}^{2}+{\text{Δ}}_{z}^{2}}\right)$ was averaged over all the brain voxels and used as measure of error, in millimeters.

### Statistical analysis

Regional mean uptake values and right-to-left asymmetry indices were calculated at VOI level for each of the images used in the construction of the tracer specific templates. The procedure to extract these values was also implemented in SAMIT for further use.

All data obtained from the VOIs and the registration error tests were analyzed using IBM SPSS Statistics 22 (SPSS Inc. Chicago, The United States). The Generalized Estimating Equations (GEE) model [17] was used to account for the repeated measurements during the analysis of the registration errors, with Gamma as distribution and Log as link function. The Quasi-likelihood under the independence model information criterion [17] was applied to find the best working correlation matrix structure applicable for the analysis, which was determined to be the independent structure (compared with auto-regressive, exchangeable, and unstructured). Wald test was used to report the *p*-values, which were considered significant for *p*<0.05.

### Voxel-based Analysis

Two voxel-based analyses were performed in SPM8, using [^11^C]PK11195 data, to evaluate the use of tracer specific templates in combination with the SAMIT package. In the first experiment Sprague-Dawley rats were studied, by comparing a control group (*n* = 11) with the focal lesion group (*n* = 10), obtained by stereotaxic injection of saline in the right corpus callosum and striatum. In the second study, Wistar rats were divided into a control group (*n* = 19) and HSE group (*n* = 14), and were scanned at day 6 or 7 after inoculation of the virus. The analysis was performed using a two-sample *t*-test design (control vs. intervention). All the images were smoothed with a 1.2 mm isotropic Gaussian kernel. The analysis was performed without global normalization, since non-specific binding of the tracer to non-activated microglia is considered to be close to zero.

For the interpretation of group differences, *T*-maps data were interrogated at *p* = 0.001 (uncorrected) and an extent threshold of 200 voxels. Only cluster with *p*<0.05 family-wise error (FWE) corrected were considered significant.

The use of the SAMIT package within SPM allows the visualization of the results over a rat ‘glass brain’ (maximum intensity projection map), and to report the coordinates in Paxinos space.

## Results

### Tracer specific PET and SPECT Templates

**Fig 2:**
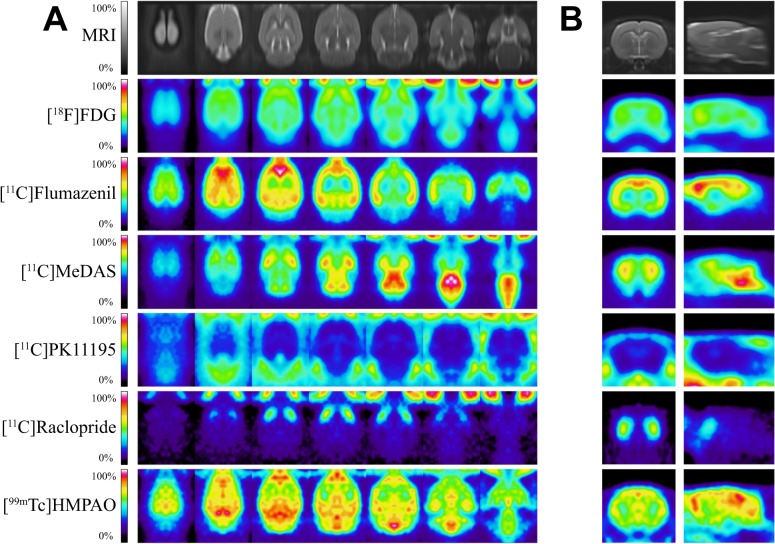
Tracer-specific PET and SPECT templates. (A) Different horizontal brain sections, and (B) sagittal and coronal sections.

Fig. 2 shows the different PET and SPECT tracer templates constructed, aligned in space with the rat stereotaxic MRI. The mean VOI uptake and right-to-left ratios, are displayed in Table 2 for [^18^F]FDG, [^11^C]flumazenil, [^11^C]MeDAS, [^11^C]PK11195, [^11^C]raclopride and [^99m^Tc]HMPAO, which were calculated from the images used in the construction of the corresponding template.

**Table 2:** Mean SUV uptake, and right/left ratio obtained using VOI analysis.

	**SUV**			**R/L ratio**				**SUV**			**R/L ratio**		
	**Mean**	*±*	**SD**	**Mean**	*±*	**SD**		**Mean**	*±*	**SD**	**Mean**	*±*	**SD**
*[* ^*18*^ *F]FDG*							*[* ^*99m*^ *Tc]HMPAO*						
Accumbens	1.06	*±*	0.05	1.00	*±*	0.06	Accumbens	0.96	*±*	0.08	1.00	*±*	0.12
Amygdala	0.78	*±*	0.04	1.02	*±*	0.04	Amygdala	0.86	*±*	0.07	1.01	*±*	0.08
Caudate-Putamen	1.28	*±*	0.06	1.00	*±*	0.02	Caudate-Putamen	0.94	*±*	0.05	1.03	*±*	0.04
Cerebellum	0.94	*±*	0.07	1.01	*±*	0.03	Cerebellum	1.05	*±*	0.07	0.98	*±*	0.05
Cortex	1.09	*±*	0.04	1.00	*±*	0.01	Cortex	1.04	*±*	0.03	0.99	*±*	0.03
Globus Pallidus	1.10	*±*	0.08	1.00	*±*	0.04	Globus Pallidus	0.87	*±*	0.09	1.02	*±*	0.17
Hippocampus	1.05	*±*	0.03	1.00	*±*	0.02	Hippocampus	1.13	*±*	0.03	1.02	*±*	0.06
Hypothalamus	0.77	*±*	0.06	1.01	*±*	0.03	Hypothalamus	1.01	*±*	0.04	1.00	*±*	0.08
Medulla	0.90	*±*	0.07	0.99	*±*	0.04	Medulla	0.89	*±*	0.07	1.01	*±*	0.07
Midbrain	1.08	*±*	0.03	1.00	*±*	0.02	Midbrain	1.10	*±*	0.04	0.99	*±*	0.05
Pons	0.85	*±*	0.10	0.98	*±*	0.03	Pons	0.91	*±*	0.05	0.99	*±*	0.07
Septum	1.05	*±*	0.04	1.00	*±*	0.03	Septum	1.01	*±*	0.09	0.96	*±*	0.08
Thalamus	1.17	*±*	0.06	1.00	*±*	0.02	Thalamus	1.07	*±*	0.03	0.99	*±*	0.06
*[* ^*11*^ *C]Flumazenil*							*[* ^*11*^ *C]MeDAS*						
Accumbens	0.63	*±*	0.15	1.01	*±*	0.18	Accumbens	1.00	*±*	0.04	0.99	*±*	0.05
Amygdala	0.66	*±*	0.13	0.97	*±*	0.07	Amygdala	0.82	*±*	0.05	1.06	*±*	0.07
Caudate-Putamen	0.71	*±*	0.14	0.99	*±*	0.04	Caudate-Putamen	1.20	*±*	0.03	1.00	*±*	0.03
Cerebellum	0.50	*±*	0.11	1.01	*±*	0.02	Cerebellum	0.85	*±*	0.05	1.04	*±*	0.02
Cortex	0.87	*±*	0.16	1.02	*±*	0.01	Cortex	0.87	*±*	0.02	1.02	*±*	0.02
Globus Pallidus	0.63	*±*	0.14	0.98	*±*	0.09	Globus Pallidus	1.28	*±*	0.05	0.99	*±*	0.04
Hippocampus	0.86	*±*	0.16	1.00	*±*	0.03	Hippocampus	0.96	*±*	0.01	1.01	*±*	0.03
Hypothalamus	0.55	*±*	0.12	1.01	*±*	0.07	Hypothalamus	1.06	*±*	0.06	0.99	*±*	0.03
Medulla	0.29	*±*	0.08	1.00	*±*	0.07	Medulla	1.20	*±*	0.04	1.00	*±*	0.06
Midbrain	0.72	*±*	0.17	1.00	*±*	0.05	Midbrain	1.31	*±*	0.05	1.01	*±*	0.02
Pons	0.34	*±*	0.09	1.01	*±*	0.05	Pons	1.40	*±*	0.05	1.01	*±*	0.02
Septum	0.68	*±*	0.14	0.98	*±*	0.07	Septum	1.07	*±*	0.05	1.01	*±*	0.06
Thalamus	0.62	*±*	0.16	1.00	*±*	0.04	Thalamus	1.21	*±*	0.04	0.99	*±*	0.03
*[* ^*11*^ *C]PK11195*							*[* ^*11*^ *C]Raclopride*						
Accumbens	0.38	*±*	0.07	0.97	*±*	0.11	Accumbens	1.50	*±*	0.53	1.01	*±*	0.11
Amygdala	0.40	*±*	0.07	1.06	*±*	0.10	Amygdala	0.94	*±*	0.35	1.03	*±*	0.14
Caudate-Putamen	0.33	*±*	0.06	0.99	*±*	0.07	Caudate-Putamen	2.37	*±*	0.83	1.01	*±*	0.03
Cerebellum	0.58	*±*	0.14	0.96	*±*	0.07	Cerebellum	0.69	*±*	0.20	1.01	*±*	0.05
Cortex	0.51	*±*	0.09	1.01	*±*	0.05	Cortex	1.02	*±*	0.32	1.06	*±*	0.06
Globus Pallidus	0.32	*±*	0.07	1.00	*±*	0.15	Globus Pallidus	2.10	*±*	0.84	0.97	*±*	0.21
Hippocampus	0.36	*±*	0.06	1.03	*±*	0.06	Hippocampus	0.86	*±*	0.26	1.00	*±*	0.08
Hypothalamus	0.39	*±*	0.07	1.00	*±*	0.11	Hypothalamus	0.86	*±*	0.29	0.97	*±*	0.14
Medulla	0.51	*±*	0.09	1.01	*±*	0.07	Medulla	0.80	*±*	0.26	1.01	*±*	0.08
Midbrain	0.38	*±*	0.08	1.01	*±*	0.10	Midbrain	0.92	*±*	0.28	1.00	*±*	0.13
Pons	0.42	*±*	0.08	1.00	*±*	0.08	Pons	0.82	*±*	0.26	1.04	*±*	0.04
Septum	0.38	*±*	0.07	1.04	*±*	0.10	Septum	1.26	*±*	0.46	0.86	*±*	0.08
Thalamus	0.36	*±*	0.07	1.01	*±*	0.05	Thalamus	1.10	*±*	0.37	0.94	*±*	0.06

The SUV values for [^18^F]FDG and [^99m^Tc]HMPAO are corrected for the mean uptake value of the whole brain.

The [^18^F]FDG uptake, expressed as SUV corrected for the mean uptake of the whole brain, was found to be fairly homogenous across the brain. The lowest relative uptake was found in the hypothalamus (0.77*±*0.06), amygdala (0.78±0.04), and pons (0.85±0.10), whereas the highest uptake was found in the caudate-putamen (1.28±0.06), thalamus (1.17±0.06) and globus pallidus (1.10±0.08). The right-to-left ratio was close to one for all regions, ranging from 0.98±0.03 in the pons to 1.02±0.04 in the amygdala.

In [^11^C]flumazenil images the lowest uptake (SUV) was found in medulla (0.29±0.08) and pons (0.34±0.09), and the highest uptake in cortex (0.87±0.16) and hippocampus (0.86±0.16). The right-to-left ratio ranged from 0.97±0.07 in the amygdala to 1.02±0.01 in the cortex.

For [^11^C]MeDAS uptake, the lowest uptake (SUV)was measured in the cerebellum (0.69±0.20), followed by the medulla (0.80±0.26), while the highest uptake was detected in the cortex (1.02±0.32) and nucleus accumbens (1.00±0.04). The right-to-left ratio was more spread than in previous tracers, with the lowest value found in the hypothalamus (0.97±0.14) and nucleus accumbens (0.99±0.05), and the highest ratios found in cortex (1.06±0.06) and amygdala (1.06±0.07).

[^11^C]PK11195 uptake (SUV)_was found to be the lowest in globus pallidus (0.32±0.05), caudate-putamen (0.33±0.06), and thalamus (0.36±0.07); while the highest uptake was found in cerebellum (0.58±0.14), cortex (0.51±0.09) and medulla (0.51±0.09). The right-to-left ratio ranged from 0.96±0.07 in the cerebellum to 1.06±0.10 in the amygdala.

For [^11^C]raclopride, the lowest uptake (SUV) was measured in the cerebellum (0.69±0.20) and medulla (0.80±0.26), and the highest uptake in the caudate-putamen (2.37±0.83) and globus pallidus (2.10±0.84). The calculated right-to-left ratios were the most spread, with the lowest ratio detected in septum (0.86±0.08) and thalamus (0.94±0.06), ranging up to the cortex (1.06±0.06) and pons (1.04±0.04).

The [^99m^Tc]HMPAO uptake (SUV) was corrected by the mean uptake of the whole brain, and was found to be lowest in the amygdala (0.86±0.07) and the globus pallidus (0.87±0.09), whereas the highest uptake was found in the hippocampus (1.13±0.03) and midbrain (1.10±0.04). The right-to-left ratios were close to one and ranged from 0.96±0.08 in the septum to 1.03±0.04 in the caudate-putamen.

### Registration errors

Table 3 summarizes the mean registration errors obtained after random misalignments of the images in relation with its original spatially normalized position. The results are expressed in millimeters and represent misregistered distances in the rat brain.

**Table 3:** Registration errors obtained after random misalignments of the images in relation with its original spatially normalized position.

				**Translate**						**Rotate**						**Scale**						**All**					
**Test**	****Strain****	****Condition****		**Mean**	±	**SD**	**Min**	**-**	**Max**	**Mean**	±	**SD**	**Min**	**-**	**Max**	**Mean**	±	**SD**	**Min**	**-**	**Max**	**Mean**		**SD**	**Min**	±	**Max**
*Standard template test*:																											
[^11^C]Flumazenil	Wistar (*n* = 13)	Tracer specific template		0.22	±	0.09	0.08	-	0.48	0.21	±	0.10	0.08	-	0.54	0.25	±	0.10	0.08	-	0.52	0.23	±	0.10	0.09	-	0.58
		FDG template		0.94	±	0.22	0.35	-	1.25	1.07	±	0.30	0.48	-	2.03	1.07	±	0.28	0.53	-	1.63	1.84	±	1.90	0.66	-	9.42
[^11^C]MeDAS	Wistar (*n* = 15)	Tracer specific template		0.89	±	0.19	0.29	-	1.21	0.84	±	0.20	0.23	-	1.19	0.92	±	0.22	0.28	-	1.29	0.86	±	0.22	0.23	-	1.29
		FDG template		1.31	±	0.33	0.60	-	2.12	1.06	±	0.32	0.46	-	2.16	1.66	±	0.48	0.69	-	3.41	1.68	±	0.90	0.61	-	6.08
[^11^C]PK11195	Wistar (*n* = 19)	Tracer specific template		0.65	±	0.27	0.16	-	1.16	0.57	±	0.24	0.16	-	1.01	0.72	±	0.32	0.18	-	1.68	0.63	±	0.29	0.17	-	2.15
		FDG template		6.77	±	4.17	0.92	-	20.18	8.47	±	3.38	1.10	-	40.91	7.61	±	5.80	0.79	-	22.69	8.77	±	5.13	1.37	-	37.14
[^11^C]Raclopride	Wistar (*n* = 6)	Tracer specific template		0.33	±	0.13	0.16	-	0.56	0.33	±	0.16	0.14	-	0.65	0.34	±	0.14	0.16	-	0.67	0.33	±	0.17	0.14	-	0.68
		FDG template		2.13	±	0.56	1.26	-	3.73	2.77	±	0.95	1.60	-	6.60	2.53	±	0.66	0.97	-	4.56	2.96	±	1.09	1.68	-	6.68
[^99m^Tc]HMPAO	Wistar (*n* = 15)	Tracer specific template		0.40	±	0.12	0.22	-	0.91	0.38	±	0.13	0.24	-	0.94	0.42	±	0.16	0.23	-	1.26	0.39	±	0.13	0.22	-	0.92
		FDG template		1.10	±	0.26	0.40	-	2.25	0.98	±	0.30	0.37	-	1.93	1.32	±	0.41	0.47	-	2.53	1.39	±	0.84	0.34	-	5.49
*Strain test*:																											
[^18^F]FDG	Sprague-Dawley (*n* = 9)	Specific		0.40	±	0.17	0.17	-	0.97	0.38	±	0.26	0.17	-	1.74	0.47	±	0.21	0.19	-	1.25	0.67	±	0.69	0.18	-	3.53
		Other		0.79	±	0.16	0.48	-	1.25	0.70	±	0.20	0.26	-	1.29	0.86	±	0.26	0.44	-	1.96	0.81	±	0.35	0.39	-	2.56
		Both		0.60	±	0.17	0.32	-	1.11	0.53	±	0.18	0.27	-	0.98	0.73	±	0.24	0.33	-	1.40	0.77	±	0.63	0.25	-	3.61
	Wistar (*n* = 25)	Specific		0.71	±	0.30	0.23	-	1.36	0.63	±	0.25	0.22	-	1.28	0.77	±	0.36	0.23	-	2.36	0.69	±	0.38	0.22	-	3.43
		Other		1.14	±	0.39	0.64	-	2.59	1.17	±	0.43	0.67	-	3.13	1.20	±	0.45	0.61	-	3.03	1.36	±	0.56	0.65	-	3.25
		Both		0.79	±	0.39	0.26	-	1.69	0.74	±	0.36	0.24	-	1.80	0.87	±	0.47	0.22	-	2.39	0.94	±	0.58	0.23	-	3.09
[^11^C]PK11195	Sprague-Dawley (*n* = 11)	Specific		0.57	±	0.33	0.19	-	1.61	0.50	±	0.33	0.25	-	1.62	0.66	±	0.36	0.27	-	1.74	0.56	±	0.36	0.22	-	1.84
		Other		0.91	±	0.21	0.40	-	1.19	0.80	±	0.19	0.34	-	1.14	1.01	±	0.28	0.30	-	1.74	0.85	±	0.25	0.33	-	1.39
		Both		0.80	±	0.22	0.31	-	1.23	0.71	±	0.21	0.26	-	1.21	0.91	±	0.29	0.28	-	1.53	0.78	±	0.29	0.22	-	1.81
	Wistar (*n* = 19)	Specific		0.63	±	0.26	0.16	-	1.16	0.58	±	0.24	0.16	-	1.00	0.71	±	0.32	0.18	-	1.92	0.63	±	0.29	0.16	-	2.07
		Other		0.96	±	0.21	0.47	-	1.47	0.92	±	0.23	0.50	-	1.53	1.08	±	0.36	0.47	-	2.75	0.94	±	0.25	0.46	-	1.66
		Both		0.65	±	0.23	0.18	-	1.05	0.59	±	0.20	0.12	-	0.97	0.72	±	0.27	0.20	-	1.68	0.65	±	0.30	0.18	-	3.03
*Size test*:																											
[^18^F]FDG	Wistar (*n* = 25)	Small image	Template same size	0.71	±	0.30	0.21	-	1.36	0.63	±	0.25	0.22	-	1.19	0.78	±	0.37	0.22	-	2.15	0.71	±	0.42	0.21	-	3.43
			Template different size	1.99	±	0.51	1.08	-	3.51	2.70	±	0.28	1.30	-	3.64	2.23	±	0.74	0.83	-	8.51	2.89	±	0.94	2.08	-	13.24
		Large image	Template same size	0.87	±	0.26	0.39	-	1.53	0.87	±	0.30	0.27	-	1.70	0.98	±	0.33	0.29	-	1.74	0.99	±	0.33	0.28	-	1.97
			Template different size	0.96	±	0.34	0.35	-	1.53	0.97	±	0.35	0.29	-	1.65	0.95	±	0.36	0.32	-	2.04	0.97	±	0.37	0.32	-	1.82
[^11^C]PK11195	Wistar (*n* = 19)	Small image	Template same size	0.65	±	0.27	0.16	-	1.16	0.57	±	0.24	0.16	-	1.01	0.72	±	0.32	0.18	-	1.68	0.63	±	0.29	0.17	-	2.15
			Template different size	3.66	±	0.68	1.06	-	4.64	4.05	±	0.12	3.78	-	4.36	3.64	±	0.67	0.96	-	4.44	3.95	±	0.44	1.95	-	5.41
		Large image	Template same size	0.62	±	0.19	0.18	-	1.16	0.72	±	0.27	0.21	-	1.46	0.78	±	0.30	0.21	-	1.90	0.76	±	0.27	0.31	-	1.64
			Template different size	0.80	±	0.30	0.20	-	1.28	0.81	±	0.30	0.20	-	1.31	0.83	±	0.31	0.20	-	1.52	0.84	±	0.30	0.18	-	1.45
*Intervention tests*:																											
[^11^C]PK11195	Sprague-Dawley	Injection of saline	Healthy (*n* = 11)	0.57	±	0.33	0.19	-	1.61	0.50	±	0.33	0.25	-	1.62	0.66	±	0.36	0.27	-	1.74	0.56	±	0.36	0.22	-	1.84
			Intervention (*n* = 10)	1.08	±	0.21	0.55	-	1.38	1.04	±	0.21	0.53	-	1.37	1.21	±	0.30	0.63	-	2.39	1.09	±	0.29	0.50	-	2.22
	Wistar	Herpes encephalitis model	Healthy (*n* = 19)	0.63	±	0.26	0.16	-	1.16	0.58	±	0.24	0.16	-	1.00	0.71	±	0.32	0.18	-	1.92	0.63	±	0.29	0.16	-	2.07
			Intervention (*n* = 14)	0.75	±	0.29	0.19	-	1.38	0.69	±	0.29	0.20	-	1.40	0.82	±	0.31	0.18	-	1.60	0.74	±	0.30	0.18	-	1.57

Units are expressed in millimeters.

To evaluate the added value of tracer specific templates, the images from healthy Wistar rats used in the construction of the templates were evaluated by comparing the registration errors obtained using a tracer specific template versus the results obtained using a “standard template” ([^18^F]FDG template). Detailed results of the GEE models can be found in Table 4. In all the misalignments tests (translation, rotation, scale, and combined) the registration errors obtained for all the tracers when registered to its own tracer specific template were statistical significant smaller than those obtained when using the “standard template” (*p*<0.001 for all the tracers). As an example, the registration errors obtained in the combined misalignment, when comparing the tracer specific template versus the “standard template”, were 0.23±0.10 vs. 1.84±1.90 for [^11^C]flumazenil, 0.86±0.22 vs. 1.68±0.90 for [^11^C]MeDAS, 0.63±0.29 vs. 8.77±5.13 for [^11^C]PK11195, 0.33±0.17 vs. 2.96±1.09 for [^11^C]raclopride, and 0.39±0.13 vs. 1.39±0.84 for [^99m^Tc]HMPAO.

**Table 4:** Registration accuracy error: tracer specific template vs. “standard template” ([^18^F]FDG template).

	**Translate**			**Rotate**			**Scale**			**Combined**		
	**B**	**95% CI**	***p-value***	**B**	**95% CI**	***p-value***	**B**	**95% CI**	***p-value***	**B**	**95% CI**	***p-value***
*[* ^*11*^ *C]Flumazenil (n = 13)*												
(Intercept)	−0.58	−0.9; −0.03	<0.001	0.07	0.01; 0.15	0.099	0.07	0.01; 0.13	0.021	0.61	0.41; 0.80	<0.001
Tracer specific template	−1.46	−1.66; −1.25	<0.001	−1.64	−1.90; −1.39	<0.001	−1.46	−1.66; −1.26	<0.001	−2.09	−2.42; −1.76	<0.001
*[* ^*11*^ *C]MeDAS (n = 15)*												
(Intercept)	0.27	0.19; 0.35	<0.001	0.05	−0.01; 0.12	0.094	0.51	0.44; 0.57	<0.001	0.52	0.41; 0.62	<0.001
Tracer specific template	−0.38	−0.48; −0.28	<0.001	−0.23	−0.33; −0.13	<0.001	−0.59	−0.68; −0.50	<0.001	−0.67	−0.81; −0.54	<0.001
*[* ^*11*^ *C]PK11195 (n = 19)*												
(Intercept)	1.91	−1.78; 2.04	<0.001	2.14	2.07; 2.20	<0.001	2.03	1.91; 2.15	<0.001	2.17	2.08; 2.26	<0.001
Tracer specific template	−2.35	−2.55; −2.15	<0.001	−2.69	−2.86; −2.53	<0.001	−2.35	−2.55; −2.16	<0.001	−2.64	−2.85; −2.42	<0.001
*[* ^*11*^ *C]Raclopride (n = 6)*												
(Intercept)	0.76	0.61; 0.90	<0.001	1.02	0.78; 1.26	<0.001	0.93	0.78; 1.08	<0.001	1.09	0.86; 1.31	<0.001
Tracer specific template	−1.85	−2.13; −1.58	<0.001	−2.14	−2.57; −1.70	<0.001	−1.99	−2.31; −1.68	<0.001	−2.18	−2.62; −1.75	<0.001
*[* ^*99m*^ *Tc]HMPAO (n = 15)*												
(Intercept)	0.10	0.04; 0.15	0.001	−0.02	−0.13; 0.08	0.667	0.28	0.20; 0.36	<0.001	0.33	0.20; 0.46	<0.001
Tracer specific template	−1.02	−1.15; −0.90	<0.001	−0.94	−1.05; −0.83	<0.001	−1.14	−1.30; −0.97	<0.001	−1.26	−1.40; −1.12	<0.001

Parameter estimates were obtained using the “standard template” as reference category.

The added value of strain specific templates was also explored using Sprague-Dawley and Wistar rats. Strain specific templates, plus a template combining the images of both strains, were tested for [^18^F]FDG and [^11^C]PK11195 (Table 5). In the GEE model, the tracer type, the strain, the template type, and the interaction of strain and template were introduced as factors. The effect of the template and the interaction of strain and template were found to be significant in all the misalignment tests (*p*<0.001), while tracer effect was found to be statistically significant only for the combined misalignment (*p* = 0.006), and the effect of the strain only for the rotation misalignment (*p* = 0.007). Using the combined misalignment as reference, the [^18^F]FDG images from Sprague-Dawley showed a registration errors of 0.67±0.69, 0.81±0.35 (*p* = 0.004), and 0.77±0.63 (*p* = 0.011) when registered to a specific strain template, opposite strain template, or a combined template respectively. With the Wistar rats the registration errors were of 0.69±0.38 for the specific template, 1.36±0.56 (*p*<0.001) for the opposite strain template, and 0.94±0.58 (*p*<0.001) for the combined template. Similarly, the [^11^C]PK11195 images from Sprague-Dawley showed a registration error of 0.56±0.36 for the strain specific template, 0.85±0.25 (*p* = 0.003) for the opposite strain template, and 0.78±0.29 (*p* = 0.01) for the combined template. For the Wistar rats the calculated registration errors were 0.63±0.29 for the specific template, 0.94±0.25 (*p*<0.001) for the opposite strain template, and 0.65±0.30 (*p*<0.001) for the combined template. In all the cases, the smallest registration errors were found when the registration was performed to the strain specific template, followed by the combined template, being the opposite strain template the one giving the largest registration errors.

**Table 5:** Registration errors: effect of strain specific template.

	**Translate**			**Rotate**			**Scale**			**Combined**		
	**B**	**95% CI**	***p-value***	**B**	**95% CI**	***p-value***	**B**	**95% CI**	***p-value***	**B**	**95% CI**	***p-value***
(Intercept)	−0.33	−0.46; −0.21	<0.001	−0.43	−0.56; −0.30	<0.001	−0.22	−0.34; −0.11	<0.001	−0.33	−0.45; −0.22	<0.001
Tracer ([^18^F]FDG)	0.03	−0.12; 0.18	0.718	0.06	−0.10; 0.21	0.471	0.01	−0.14; 0.16	0.916	0.20	0.06; 0.35	<0.001
Strain (Sprague-Dawley)	−0.02	−0.19; 0.15	0.828	−0.06	−0.24; 0.13	0.551	0.03	−0.13; 0.20	0.693	0.00	−0.16; 0.16	0.982
Template (same strain)	−0.08	−0.13; −0.02	0.012	−0.10	−0.17; −0.04	0.003	−0.08	−0.15; −0.02	0.010	−0.18	−0.26; −0.11	<0.001
Template (different strain)	0.37	0.28; 0.46	<0.001	0.45	0.35; 0.55	<0.001	0.36	0.27; 0.44	<0.001	0.37	0.29; 0.46	<0.001
Strain * Template same strain	−0.28	−0.50; −0.07	0.009	−0.24	−0.48; 0.01	0.059	−0.29	−0.48; −0.09	0.004	−0.06	−0.29; 0.16	0.581
Strain * Template different strain	−0.19	−0.29; −0.08	0.001	−0.27	−0.39; −0.15	<0.001	−0.23	−0.33; −0.13	<0.001	−0.31	−0.41; −0.20	<0.001

The test was performed with Sprague-Dawley, [^18^F]FDG (*n* = 9) and [^11^C]PK11195 (*n* = 11); and Wistar rats, [^18^F]FDG (*n* = 25) and [^11^C]PK11195(*n* = 19). Parameters estimates were obtained using [^11^C]PK11195, Wistar, and combined template as reference categories.

In addition, the relevance of the image and template size in the registration errors was explored using [^18^F]FDG and [^11^C]PK11195 images from Wistar rats (Table 6). In the GEE model, the tracer type, the image size, and template size were included as factors. In addition, the interaction between tracer and image size, tracer and template size, and image and template sizes, were included in the model. For all the misalignments, all factors and interactions were found to be statistically significant (*p*<0.01) with the exception of the factor ‘tracer’ that were not significant in any of the models. For the combined misalignment, the registration errors obtained with [^18^F]FDG using the ‘small images’ were 0.71±0.42 when registered to the template of the same size, and 2.89±0.94 when registered to a larger size (*p*<0.001). For the ‘large images’ the obtained error was 0.99±0.33 for the registration to a template of the same size, and 0.97±0.37 for the registration to the small template (*p* = 0.056). For [^11^C]PK11195 tracer, the registration errors of the small images were of 0.63±0.29 when the registration was done to the template of the same size, and 3.95±0.44 when registered to the larger template (*p*<0.001). The registration error of the large image to the template of the same size was 0.76±0.27, while the registration error to the small template gave an error of 0.84±0.30 (*p* = 0.002).

**Table 6:** Registration errors: effect of the template size.

	**Translate**			**Rotate**			**Scale**			**Combined**		
	**B**	**95% CI**	***p-value***	**B**	**95% CI**	***p-value***	**B**	**95% CI**	***p-value***	**B**	**95% CI**	***p-value***
(Intercept)	−0.14	−0.30; −0.01	0.075	−0.13	−0.29; 0.02	0.092	−0.12	−0.27; 0.03	0.126	−0.13	−0.28; 0.02	0.085
Tracer ([^18^F]FDG)	0.04	−0.17; 0.25	0.696	0.05	−0.16; 0.26	0.649	0.02	−0.18; 0.22	0.844	0.07	−0.13; 0.27	0.517
Image size (small)	1.36	1.20; 1.53	<0.001	1.47	1.30; 1.64	<0.001	1.34	1.19; 1.50	<0.001	1.46	1.30; 1.63	<0.001
Template size (same size)	−0.41	−0.53; −0.29	<0.001	−0.26	−0.38; −0.13	<0.001	−0.20	−0.33; −0.07	0.002	−0.19	−0.30; -0.07	0.001
Tracer * Image size	−0.52	−0.74; −0.30	<0.001	−0.33	−0.56; −0.11	0.004	−0.39	−0.60; -0.18	<0.001	−0.30	−0.52; −0.08	<0.001
Tracer * Template size	0.44	0.28; 0.59	<0.001	0.26	0.11; 0.41	0.001	0.33	0.18; 0.48	<0.001	0.28	0.13; 0.42	<0.001
Image size * Template size	−1.17	−1.33; −1.01	<0.001	−1.56	−1.71; −1.41	<0.001	−1.28	−1.43; -1.13	<0.001	−1.56	−1.71; −1.42	<0.001

The test was performed with Wistar rats, using [^18^F]FDG (*n* = 25) and [^11^C]PK11195 (*n* = 19). Parameters estimates were obtained using [^11^C]PK11195, with large image size and large template size as reference categories.

The registration error was also explored in two different disease models, using [^11^C]PK11195 as the tracer; the first one with a focal lesion caused by stereotaxic saline injection in the brain, and the other one based on the HSE model, which is known to cause a broader alteration in brainstem uptake. In both cases the GEE model was estimated using ‘group’ (intervention vs. healthy) as factor (Table 7). In the focal lesion model, for all the misalignment tests the registration error was found to be higher in the lesion group than in the healthy group (*p*<0.005). For the combined misalignment the registration error in healthy rats was 0.56±0.36, while for the images of the rats with the focal lesion the error was 1.09±0.29 (*p* = 0.001). Contrary, in the images obtained from the HSE model, the registration error was not found to be statistically significant different in any of the misalignment tests between healthy and HSE rats (e.g. 0.63±0.29 vs. 0.74±0.30, *p* = 0.372, for the combined misalignment).

**Table 7:** Registration errors: effect of an intervention.

	**Translate**			**Rotate**			**Scale**			**Combined**		
	**B**	**95% CI**	***p-value***	**B**	**95% CI**	***p-value***	**B**	**95% CI**	***p-value***	**B**	**95% CI**	***p-value***
*Focal lesion*:												
(Intercept)	−0.56	−0.89; −0.22	0.001	−0.69	−1.08; −0.30	<0.001	−0.42	−0.71; −0.12	0.005	−0.57	-0.93; −0.22	0.002
Intervention group	0.63	0.20; 1.06	0.004	0.73	0.25; 1.21	0.003	0.61	0.24; 0.97	0.001	0.66	0.21; 1.11	0.004
*HSE*:												
(Intercept)	−0.46	−0.64; −0.28	<0.001	−0.55	−0.73; −0.37	<0.001	−0.35	−0.52; −0.18	<0.001	−0.46	−0.64; −0.28	<0.001
Intervention group	0.17	−0.16; 0.49	0.321	0.19	−0.16; 0.53	0.295	0.15	−0.14; 0.43	0.312	0.15	−0.18; 0.48	0.368

The test were performed with [^11^C]PK11195 images. For the focal lesion test, Sprague-Dawley rats were divided in a healthy control group (*n* = 11) and intervention group (*n* = 10). For the herpes encephalitis (HSE) model, Wistar rats were divided in a healthy group (*n* = 19) and intervention group (*n* = 14). Parameter estimates were obtained using the healthy groups as reference category.

### Voxel-based analysis of disease models

**Fig 3:**
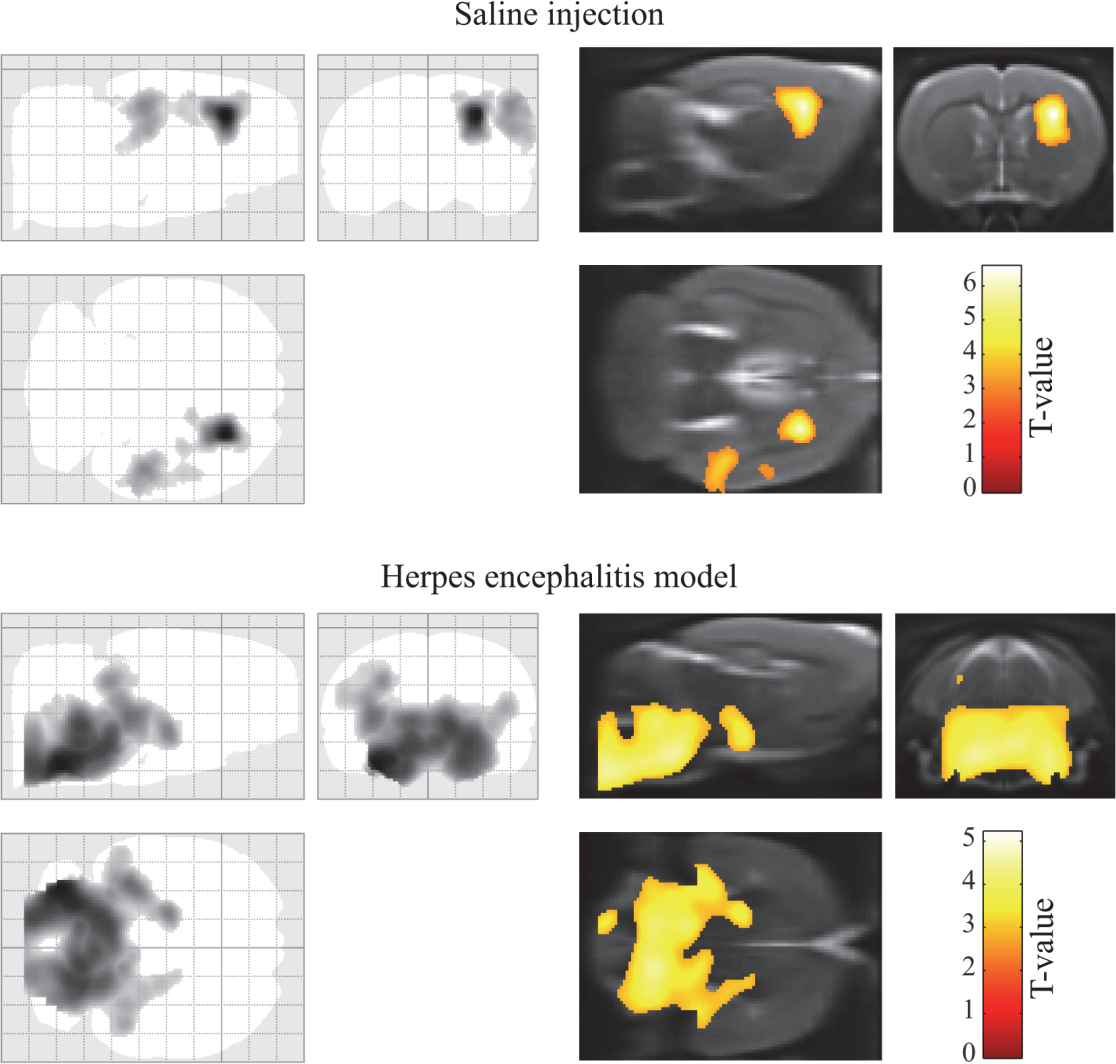
Voxel-based analysis. Statistically significant (p<0.05 family-wise error corrected at cluster level) increased uptake of [^11^C]PK11195 was found in lesion groups as compared with control animals. In the top section, the location of the stereotaxic injection of saline is clearly defined in the right corpus callosum and caudate-putamen. In the lower section of the figure, the results from the herpes encephalitis model showed a clear inflammatory process in the brainstem.

The results of the voxel-based analysis are shown in Fig. 3, and summarized in Table 8. In the focal lesion a statistically significant increase of [^11^C]PK11195 (*p* = 0.048 FWE corrected at cluster level) was found in the lesioned rats involving the right caudate-putamen, and corpus callosum, with the maximum peak located at the right caudate-putamen (*x*,*y*,*z* = 3.3,0,-3.4). For the HSE model, a statistically significant increase in uptake of [^11^C]PK11195 (*p*<0.001 FWE corrected at cluster level) was found in medulla and pons bilaterally, with maximum uptakes located in the left medulla (*x*,*y*,*z* = -3.8,-11.8,-9.6) and left pons (*x*,*y*,*z* = -2.0,-9.2,-8.8).

**Table 8:** Voxel-based analysis.

	**Cluster-level**			**Peak-level**				
	**FWE corr.**	**uncorr.**	**voxels**	**FWE corr.**	**uncorr.**	**Coordinates**		
						**x**	**y**	**z**
Focal lesion	0.048	0.072	883	0.006	<0.001	3.3	0	−3.4
Herpes encephalitis	<0.001	<0.001	15747	0.017	<0.001	−3.8	−11.8	−9.6
				0.068	<0.001	−2.0	−9.2	−8.8
				0.072	<0.001	1.5	−10.4	−7.2

Increased uptake of [^11^C]PK11195 in the intervention group as compared to the healthy rats. For the focal lesion test, Sprague-Dawley rats were divided in a healthy control group (*n* = 11) and rats stereotaxic injected with saline (*n* = 10). For the herpes encephalitis model, Wistar rats were divided in a healthy group (*n* = 19) and infected rats (*n* = 14). For the interpretation of group differences, *T*-maps data were interrogated at *p* = 0.001 (uncorrected) and an extent threshold of 200 voxels. Only cluster with *p*<0.05 family-wise error (FWE) corrected were considered significant.

## Discussion

The registration of individual images to a corresponding reference template is a crucial step prior to voxel-wise data comparison and greatly facilitates analyses with predefined regions of interest. While for radionuclide data the optimal procedure would be to utilize individual MRI scans for each animal, dedicated (ultra-high field) animal MRI systems or clinical scanners together with specific coils [18] are not easily accessible for many research groups. Two other alternatives remain: inter-modality spatial normalization of the functional images to an MRI template or intra-modality spatial normalization to a functional template. Intra-modality spatial normalization was found to provide significant lower misregistration errors than normalization to a MRI template. This strengthens the use of customized PET templates for spatial normalization [19]. Therefore, we constructed and validated tracer specific templates for rat brain studies with a variety of ligands targeting different aspects of the brain physiology in animal models.

The construction of these tracer specific PET and SPECT templates was performed using healthy adult male Wistar rats. These templates were aligned with a widely used stereotaxic T_2_-weighted MRI template for the rat brain [4], which is co-registered with the Paxinos and Watson anatomical atlas [20]. The use of this reference MRI permits the report of results directly in coordinates corresponding to the Paxinos space, as well as the definition of VOI structures based on the same atlas. Moreover, the MRI template is accompanied by tissue class distribution maps (brain, cerebrospinal fluid, muscle and other tissue) that can be used for segmentation analysis or partial volume correction.

In our setup, the validity of the templates was assessed by evaluating the individual images used for the construction of the templates, the residual registration error obtained from the images after the application of a random misalignment, and the feasibility to use the templates in a voxel-based analysis.

Although the SUV quantification in each of the defined regions using VOI measurements, and the right-to-left ratio cannot be used by itself to determine the feasibility of the templates, it can give us an insight into the characteristics of the images that were used for the construction of the specific templates. In our work, the relative standard deviation between images in each of the defined region was relatively low with a mean variation of 18% (1–40%), while the right-to-left ratios presented an even smaller mean variation of 7% (1–21%). This variability is expected as consequence of the individual differences in tracer uptake, as well as different physiological conditions and measurement errors.

Based on the residual registration errors the use of the templates for the spatial normalization of small animal PET and SPECT brain data was evaluated. These errors were obtained from the measurement of the distance between the original voxel position of the image and its recovered position after a random misalignment, averaged over all voxels. This procedure, integrated in the SAMIT package, was used under several conditions or tests to explore the added value of tracer specific and strain specific templates. In the first of these tests, the [^18^F]FDG template was chosen as the “standard template”, since it is the most frequently used tracer and its template is generally distributed in neuroimaging software packages. For all the tracers, performance of the registration was significantly better when the tracer specific template was used while higher registration errors were obtained with the “standard template” (*p*<0.001). One clear case of this added value was found with [^11^C]PK11195 images where, for example, the mean residual registration error for combined misalignments was 0.63±0.29 mm (with a maximum error of 2.15 mm) using the tracer specific template, and the registration to the “standard template” was 8.77±5.13mm (with a maximum error of 37.14 mm). In addition, the possibility to have differences in the tracer uptake between rat strains, and consequently in the performance of the template, was also evaluated with [^18^F]FDG and [^11^C]PK11195 images. The smallest registration errors were obtained when the images were registered to its own strain specific template (0.59±0.33 mm), followed by the use of a template that combines both rat strains (0.75±0.38 mm). The largest errors were found when the images were registered to the template of opposite strain image data (1.00±0.35 mm).

The differences in the registration errors between healthy rats and lesioned rats were also evaluated. The mean registration error obtained from the misalignments of images of healthy rats was 0.53±0.20 mm (range: 0.08–3.43 mm), while in the lesion models this error tended to be higher. However, only for the focal lesion model differences were found to be statistically significant (1.10±0.25, *p*<0.005).

Finally, the effect of the image size and template size was explored for [^18^F]FDG and [^11^C]PK11195 images. The smallest registration errors were found when image data and template had the same size (*p*≤0.001), and especially when both image and template had a small size (*p*<0.001).

Overall, the results obtained in the present study, indicate that the use of strain and tracer specific templates is the most appropriate approach when performing the spatial normalization of PET and SPECT functional rat brain images. Additionally, it is advisable to have images with the same dimensions as those of the reference template. When considering the use of tracer specific templates, it is important to realize that the microPET Focus 220 used in the current study has a resolution of ≤1.4 mm at the center of the field of view, and that the spatial resolution that can be achieved by using the U-SPECT-II SPECT camera with the 75 focused pinholes collimator is around 0.8 mm. Thus, the mean registration errors overall found in this study were smaller than the spatial resolution of the cameras, and these results are in agreement with human literature data (e.g., when considering relative values based on image resolution: 1.1–2.4 mm accuracy for PET and 1.6–2 mm SPECT devices [21]).

Furthermore, we presented and evaluated the integration of the constructed templates with the SAMIT package for performing a voxel-based analysis in SPM. [^11^C]PK11195 images of two different models were explored for this purpose. In the first test, a focal lesion model was chosen to evaluate the accuracy of the coordinates of a known inflammatory process induced by stereotaxic injection of saline in the rat brain. The obtained results showed a significant increased uptake of the tracer in the intervention group as compared with a healthy group, in the region of the corpus callosum and caudate-putamen; and the reported coordinates are consistent with the location where the lesion was induced [8]. Also, a broader inflammatory process was explored by using the herpes encephalitis model, which is know to produce a microglial activation in the brainsteam at 6–7 days after virus inoculation [9]. A statistical significant increase in the [^11^C]PK11195 uptake was detected bilaterally in the brainstem of the intervention group, with the highest increase located in the left side of pons and medulla.

Although in the present study the methodology for the construction of tracer specific templates [2,3] was validated and additional tests were performed under different conditions (such as the use of a “standard template”, use of two different rat strains, and the comparison of two templates and images sizes), it would be of interest to further evaluate the performance of the templates for functional imaging of other disease models, with different tracers, and even comparing alternative algorithms for spatial normalization. Also, while this same methodology was proved to be valid also in mice [3], it would be of interest to perform similar tests with other animal strains. However, it seems that the presented approaches are the most appropriate for those studies where there are no CT or MRI data of a hybrid microPET/CT or microPET/MRI system available (which most probably will allow a more robust normalization procedure, less dependent on the tracer uptake pattern or disease state of the animals).

There are other commercial packages such as PMOD which offers [^18^F]FDG templates for rat [22]. However, as has been demonstrated, the construction of tracer specific templates is extremely relevant and the normalization of the broad variety of tracer data cannot be performed only by means of MRI or [^18^F]FDG templates. This is especially relevant for those tracers where the binding does not reflect any substantial anatomical information that can be used for inter-modality techniques.

Moreover, we have presented the integration of the tracer specific templates and the SAMIT package within the widely used SPM environment. We have also tested the templates with other popular functional imaging packages, i.e. FSL and AFNI, obtaining very consistent results (not presented). As with other functional and structural imaging templates, the tracer specific templates presented here can be easily integrated within any other similar packages.

## Conclusion

In conclusion, the present work shows that the construction of PET and SPECT strain and tracer specific templates is a promising and sensitive tool in the evaluation of human brain diseases through the use of specific rat models. Moreover, the current methodology for the construction and validation of the templates is a reliable approach for the design of further specific templates. This procedure can be easily replicated for the construction of other tracer specific templates, according to the needs of each individual research group. The templates and the SAMIT toolbox, together with all the code used in this work, will be available for the research community.

The use of PET and SPECT rat brain templates, aligned in space with the stereotaxic Paxinos coordinate space, allows accurate registration of functional rat brain data, using automatic registration algorithms available in standard packages (e.g., SPM, FSL), and subsequent analysis based on predefined volumes of interest and/or voxel-based approaches. The low intersubject variability and the low registration errors obtained, comparable to those observed in analogous processing of human data, suggest that the constructed tracer specific templates can be used for the precise study of interventional or longitudinal studies in the rat brain.
