# Correction: A Standardized Method for the Construction of Tracer Specific PET and SPECT Rat Brain Templates: Validation and Implementation of a Toolbox

**DOI:** 10.1371/journal.pone.0143900

**Published:** 2015-11-24

**Authors:** David Vállez Garcia, Cindy Casteels, Adam J. Schwarz, Rudi A. J. O. Dierckx, Michel Koole, Janine Doorduin

There are errors in the Abstract of the manuscript. Several “&” symbols appear throughout. The correct symbol should be “<”.

There is an error in the “Registration Error” subsection, which is within the “Materials and Methods” section. The third to last sentence of that subsection should read: Each resultant image volume was smoothed with a Gaussian kernel of 0.8mm and then registered again to the selected template with affine registration using least squares function.
